# The subcellular distribution and function of MTA1 in cancer differentiation

**DOI:** 10.18632/oncotarget.2095

**Published:** 2014-06-11

**Authors:** Jian Liu, Dongkui Xu, Haijuan Wang, Ying Zhang, Yanan Chang, Jinlong Zhang, Jia Wang, Chunxiao Li, Huan Liu, Mei Zhao, Chen Lin, Qimin Zhan, Changzhi Huang, Haili Qian

**Affiliations:** ^1^ State Key Laboratory of Molecular Oncology; Cancer Institute/Hospital, Peking Union Medical College & Chinese Academy of Medical Sciences, Beijing, China; ^2^ Medical Research Center, Beijing ChaoYang Hospital, Capital Medical University, Beijing, China; ^3^ Department of Abdominal Surgery, Cancer Institute/Hospital, Peking Union Medical College & Chinese Academy of Medical Sciences, Beijing, China; ^4^ Department of Gynecology Minimally Invasive Center, Beijing Obstetrics and Gynecology Hospital, Capital Medical University, Beijing, China

**Keywords:** MTA1, Subcellular distribution, Cancer, Differentiation

## Abstract

The functions and mechanisms of metastasis-associated protein 1 (MTA1) in cancer progression are still unclear due to a lagged recognition of the subcellular localization. In the present study, using multiple molecular technologies we confirmed for the first time that MTA1 localizes to the nucleus, cytoplasm and nuclear envelope. MTA1 is primarily localized in the nucleus of normal adult tissues but in the cytoplasm of embryonic tissues. While in colon cancer, both distributions have been described. Further investigation revealed that MTA1 localizes on the nuclear envelope in a translocated promoter region (TPR)-dependent manner, while in the cytoplasm, MTA1 shows an obvious localization on microtubules. Both nuclear and cytoplasmic MTA1 are associated with cancer progression. However, these functions may be associated with different mechanisms because only nuclear MTA1 has been associated with cancer differentiation. Overexpression of MTA1 in HCT116 cells inhibited differentiation and promoted proliferation, whereas MTA1 knockdown resulted in cell differentiation and death. Theses results not only suggest that nuclear MTA1 is a good marker for cancer differentiation diagnosis and a potential target for the treatment of cancers but also reveal the necessity to differentially examine the functions of nuclear and cytoplasmic MTA1.

## INTRODUCTION

Metastasis-associated protein 1 (MTA1) was initially identified as a candidate metastasis-associated gene through differential cDNA library screening techniques using the 13762NF rat mammary adenocarcinoma metastatic system[1-3]. In addition to breast cancer, MTA1 was also proved to be closely correlated with aggressiveness in most types of human cancers[4]. Although the contribution of MTA1 to the promotion of tumor invasion and metastasis has been well characterized, a role for this protein in other malignances, such as cancer differentiation, has remained largely unexplored. Moreover, the underlying mechanism of MTA1 in cancer promotion remains obscure; the only well-studied mechanism is the function of MTA1 in the nucleus with other components of the nucleosome remodeling deacetylase (NuRD) complex to repress gene transcription [5-7]. However, a role for MTA1 independent of NuRD has also been reported [8-12]. Thus, it is important to explore other possible mechanisms by which MTA1 promotes cancer progression.

In cells, protein function relies on proper positioning and correct cooperation with copartners. Localization information will provide valuable clues concerning the function and underlying mechanism of a protein. The subcellular distribution of MTA1 in cells has been poorly studied. Sequence analysis of the primary structure shows multiple DNA-binding motifs and nuclear localization signals in the MTA1 protein[4, 13, 14], indicating the probable localization of MTA1 in the nucleus for DNA binding. Indeed, fusion-expression tag fluorescence tracing initially indicated that MTA1 was exclusively localized in the nucleus [15, 16]. However, subsequent studies have also observed the distribution of MTA1 in the cytoplasm[17-19]. Thus, there is much controversy concerning the subcellular distribution of MTA1, particularly regarding whether MTA1 indeed has cytoplasmic distribution. Until recently, no systematic experiments have been conducted to resolve this question.

The present study aimed to explore the potential role and mechanism of MTA1 in cancer. We first identified the expression and distribution pattern of MTA1 using multiple molecular technologies, including immunohistochemistry, cell immunofluorescence, GFP tag tracking, Western blot analysis, immunoprecipitation, in situ proximity ligation assay (PLA), and immuno-electron microscopy. And then by colon cancer microarray analyses, we found a novel role of MTA1 in inhibiting cancer differentiation in the nucleus, and proposed that the nuclear and cytoplasmic components of MTA1 may drive cancer progression through different mechanisms.

## RESULTS

### MTA1 primarily localizes to the nucleus in most normal adult tissues

**Figure 1:**
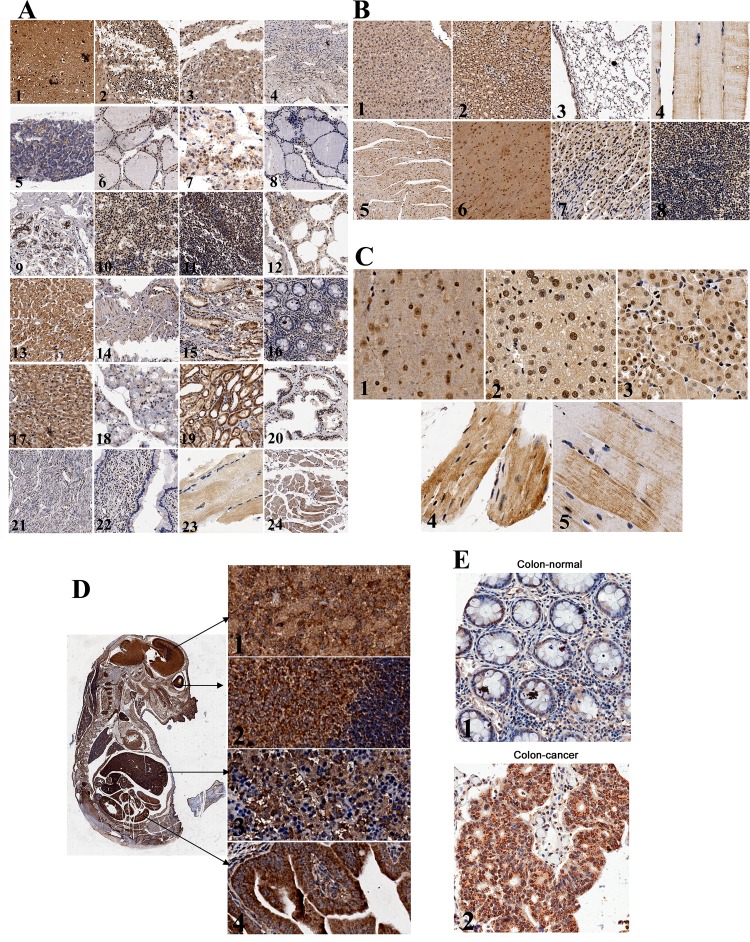
Expression and localization of endogenous MTA1 in tissues detected using immunohistochemistry A, Expression of MTA1 in normal adult human tissues: 1. brain, 2. cerebellum, 3. adrenal gland, 4. ovary, 5. pancreas, 6. parathyroid gland, 7. pituitary gland, 8. thyroid, 9. mammary gland, 10.spleen, 11. tonsil, 12. lung, 13. cardiac muscle, 14. esophagus, 15. stomach, 16. colon, 17. liver, 18. salivary gland, 19. kidney, 20. prostate, 21. Endometrium, 22. cervix, 23. skeletal muscle, and 24. throat; B, Expression of MTA1 in normal adult mouse tissues: 1. liver, 2. kidney, 3. lung, 4. skeletal muscle, 5. cardiac muscle, 6. brain, 7. stomach, and 8. spleen; C, Subcellular distribution of MTA1: 1. brain, 2. liver, 3. kidney, 4. cardiac muscle, and 5. skeletal muscle; D, Left, expression of MTA1 in the mouse embryo (day 14); Right, subcellular localization of MTA1 in original tissues: 1. brain, 2. eyes, 3. liver, and 4. intestines; E, Expression and subcellular localization of MTA1 in normal (1) and cancerous (2) colon tissues.

To determine the expression and localization of MTA1, we immunohistochemically stained for endogenous MTA1 in adult mouse and human normal tissues. In the 24 human adult tissues and 8 mouse adult tissues examined, MTA1 was expressed in all normal tissues, although the expression levels greatly differed. In general, low levels of MTA1 were expressed in most adult normal tissues, except the brain, liver, kidney, and cardiac muscle (Fig. 1A, 1B), indicating that MTA1 may play important physiological roles in these tissues.

We also observed that MTA1 localized to both the nucleus and cytoplasm and accumulated in the nucleus in most adult normal tissues (Fig. 1C, 1-3), which is consistent with previous studies[19]. However, in cardiac and skeletal muscle, MTA1 staining was primarily detected in the cytoplasm, whereas the nucleus was barely stained (Fig. 1C, 4 and 5). These results have not been previously reported.

### MTA1 localizes mainly at cytoplasm of embryonic tissues

The expression of MTA1 in mouse embryos was also detected. In general, MTA1 shows relatively higher expression throughout the entire embryo, and MTA1 expression is particularly high in nerve tissues, such as the brain, eyes, and spinal cord (Fig. 1D). Interestingly, in contrast with most adult tissues, we observed that the majority of MTA1 was localized to the cytoplasm in the original developmental tissues, such as the brain, eyes, liver, and intestines, etc. (Fig. 1D, 1-4).

### Higher MTA1 expression was detected in the nucleus and cytoplasm in colon cancer tissues

Because MTA1 is up-regulated in cancers, we also investigated the distribution of MTA1 in colon cancer tissues. Similar to normal colon tissues, MTA1 localizes to both the nucleus and cytoplasm in colon cancers (Fig. 1E and Fig. 7). In the colon cancer tissue microarray analysis shown below, MTA1 was stained at a higher level in the nucleus in 22.76% of cancer tissues and at a higher level in the cytoplasm in 18.28% of cancer tissues.

The immunohistochemistry results demonstrated that MTA1 localized to both the nucleus and cytoplasm; however, initial reports using indirect immunofluorescence (IF) or GFP-tag tracing have indicated that MTA1 localizes exclusively in the nucleus[16, 20]. Therefore, to resolve this question, we probed MTA1 using a monoclonal antibody or GFP-tag and detected the subcellular distribution of MTA1 in cells using fluorescence microscopy.

### Endogenous MTA1 localized to both the nucleus and cytoplasm, and apparent nuclear envelope MTA1 localization was also detected in some cell lines using IF

**Figure 2:**
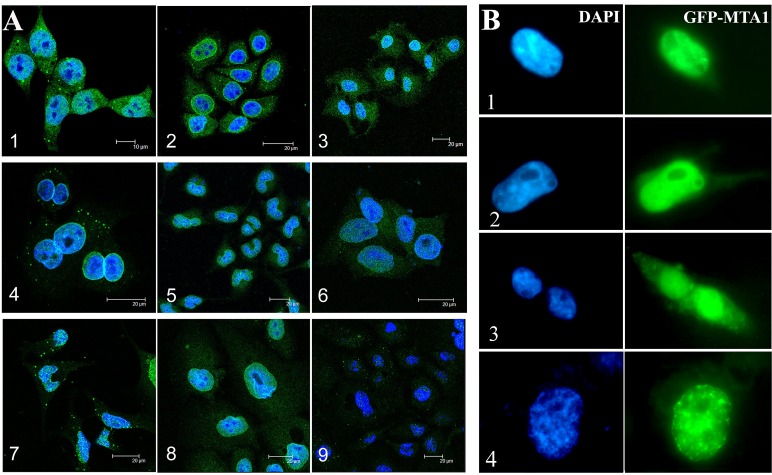
Subcellular localization of MTA1 in cell lines detected through IF (A) and GFP-tag tracing (B). A, 1. HCT116, 2. NCI-H446, 3. Ishikawa, 4. SF-767, 5. Hep3B, 6. HCT8, 7. HEK293, 8. HaCaT, and 9. Caski; B, Distributions of GFP-MTA1 expression in HEK293 cells. 1 and 2 show cells with low GFP-MTA1 expression in the cytoplasm, 3. cells with high cytoplasmic GFP-MTA1 expression, 4. a cell with apparent GFP-MTA1 fluorescence on the nuclear envelope.

To determine the distribution of MTA1, nine human cell lines, including both cancer and normal cell lines, were immuno-stained using a mouse monoclonal antibody. Consistent with the immunohistochemistry results, IF analysis also showed that MTA1 was expressed in both the nucleus and cytoplasm of all of the examined cell lines (Fig. 2A). Although the majority of MTA1 accumulated in the nucleus, we also observed obvious cytoplasmic MTA1 fluorescence, particularly in HCT116, NCI-H446, and Ishikawa cells. Interestingly, we also observed apparent MTA1 fluorescence in the nuclear envelope of NCI-H446, SF-767, Hep3B, and HaCaT cells (Fig. 2A, 2, 4, 5, 8), which is consistent with the immunogold electron microscopy data (Fig. 5A). The localization of MTA1 to the nuclear envelope has not been previously reported.

### The localization of MTA1 in the nucleus, cytoplasm, and nuclear envelope was verified through GFP-tag tracing

To verify the results described above, we transfected HEK293 cells with the GFP-tagged vector pEGFP-MTA1 and traced the distribution of exogenous MTA1. We detected cytoplasmic MTA1-GFP fluorescence in virtually all transfected cells, although the fluorescence intensity greatly fluctuated. Moreover, we observed that about 10% of cells showed strong fluorescence in the cytoplasm and observed a few cells with obvious nuclear envelope fluorescence (Fig. 2B). For control pEGFP-C2 vectors, GFP showed diffuse expression across the entire cell. These results provide further support for MTA1 localization in the nucleus, cytoplasm, and nuclear envelope, suggesting that the localization of MTA1 may be associated with the state of the cell.

### Western blot technology confirmed the nuclear and cytoplasmic distribution of MTA1

**Figure 3:**
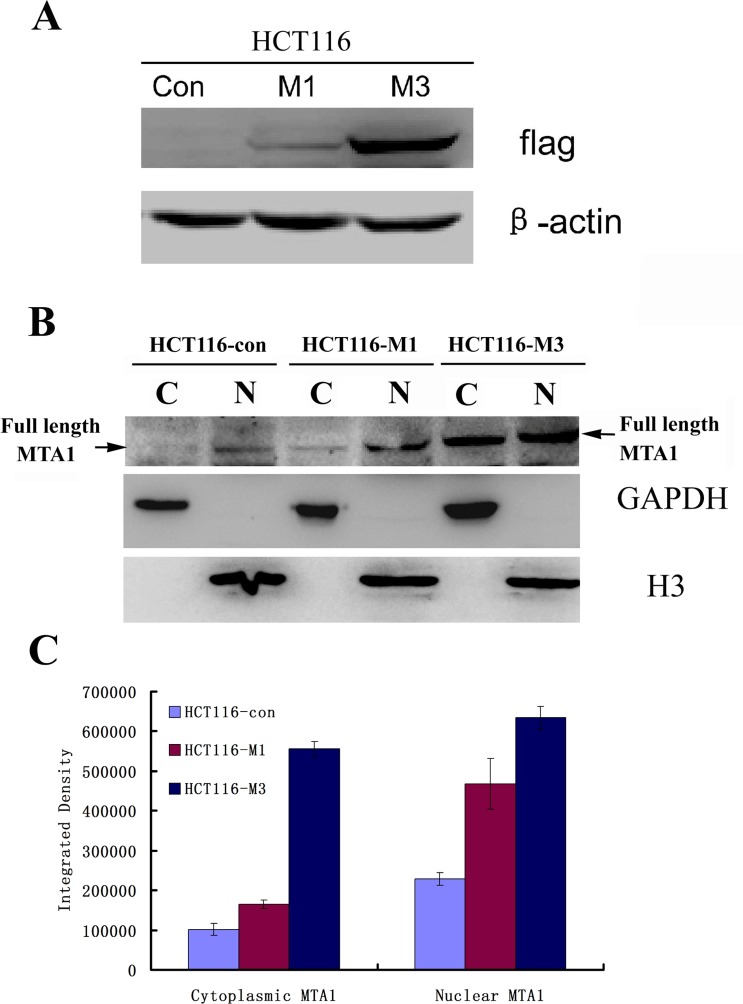
Both nuclear and cytoplasmic MTA1 are enhanced with the increasing expression of exogenous MTA1 in HCT116 cells A, Western blot analysis of the different levels of flag-tagged MTA1 expression in stably transfected overexpressing HCT116 cell clones, HCT116-M1 and HCT116-M3, using an anti-Flag antibody. B, The expression of full-length MTA1 to the cytoplasm (C) and nucleus (N) were detected through Western blot analysis using an anti-MTA1 antibody. C, Quantitive analysis of the nuclear and cytoplasmic MTA1 in B using Image J software. Results represent the mean±S.D. of triplicate experiments.

To further confirm the MTA1 distribution, we extracted the nuclear and cytoplasmic cell fractions and analyzed the expression of MTA1 in each fraction. As shown in Figure 3, the full-length 80 kDa MTA1 localized to both the nucleus and cytoplasm in control cells (HCT116-con) or stably transfected MTA1-overexpressing cells (HCT116-M1 and HCT116-M3) (Fig. 3A and 3B). Moreover, both nuclear and cytoplasmic MTA1 correspondingly increases with increasing exogenous MTA1 expression (Fig. 3B and 3C). This result further confirmed that MTA1 localizes to both the nucleus and cytoplasm.

### MTA1 interacts with HDAC2 in both the nucleus and cytoplasm

**Figure 4:**
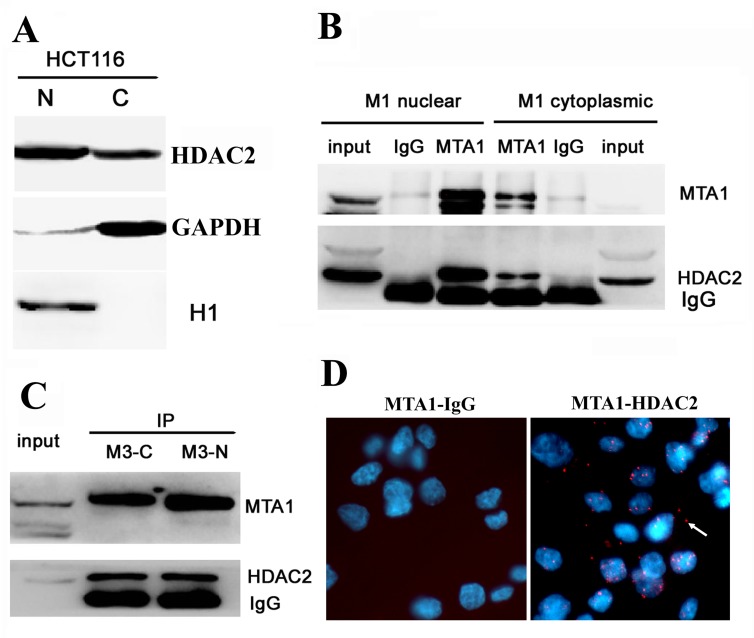
MTA1 interacts with HDAC2 at both the nucleus and cytoplasm A, Western blot analysis showing the localization of HDA2 to both the nucleus (N) and cytoplasm (C) of HCT116 cells; B, In vitro immunoprecipitation analysis of the MTA1-HDAC2 interaction in the nuclear and cytoplasmic fractions of HCT116-M1 cells; C, In vitro immunoprecipitation analysis of the MTA1-HDAC2 interaction in the nuclear and cytoplasmic fractions of HCT116-M3 cells; D, In situ PLA analysis. left, the control interaction detected using mouse antibody against MTA1 and rabbit IgG; right, in situ PLA visualization of the MTA1-HDAC2 interaction in both the nucleus and cytoplasm of HCT116 cells. The white arrow shows the positive signal in the cytoplasm.

Histone deacetylase 2 (HDAC2) interacts with MTA1 in the NuRD complex, which mediates chromatin deacetylation and inhibits gene transcription in the nucleus. We also observed that HDAC2 was localized to both the nucleus and cytoplasm in HCT116 cells (Fig. 4A). Therefore, we were wondered whether MTA1 and HDAC2 also interacted in the cytoplasm. Thus, we extracted both the cytoplasmic and nuclear fractions from HCT116-M1 and HCT116-M3 cells and detected the interaction between MTA1 and HDAC2 through in vitro IP, followed by Western blot analysis. As shown in Figures 4B and 4C, the in vitro IP analysis showed that MTA1 interacts with HDAC2 in both the nucleus and cytoplasm in both cell lines, and the strength of this interaction increased with increasing cytoplasmic MTA1 expression in HCT116-M3 cells. In situ PLA technology was also performed to visualize the localization of the MTA1/HADC2 interaction. As shown in Figure 4D, about 1/4 of the total positive signals were observed in the cytoplasm of HCT116 cells.

**Figure 5:**
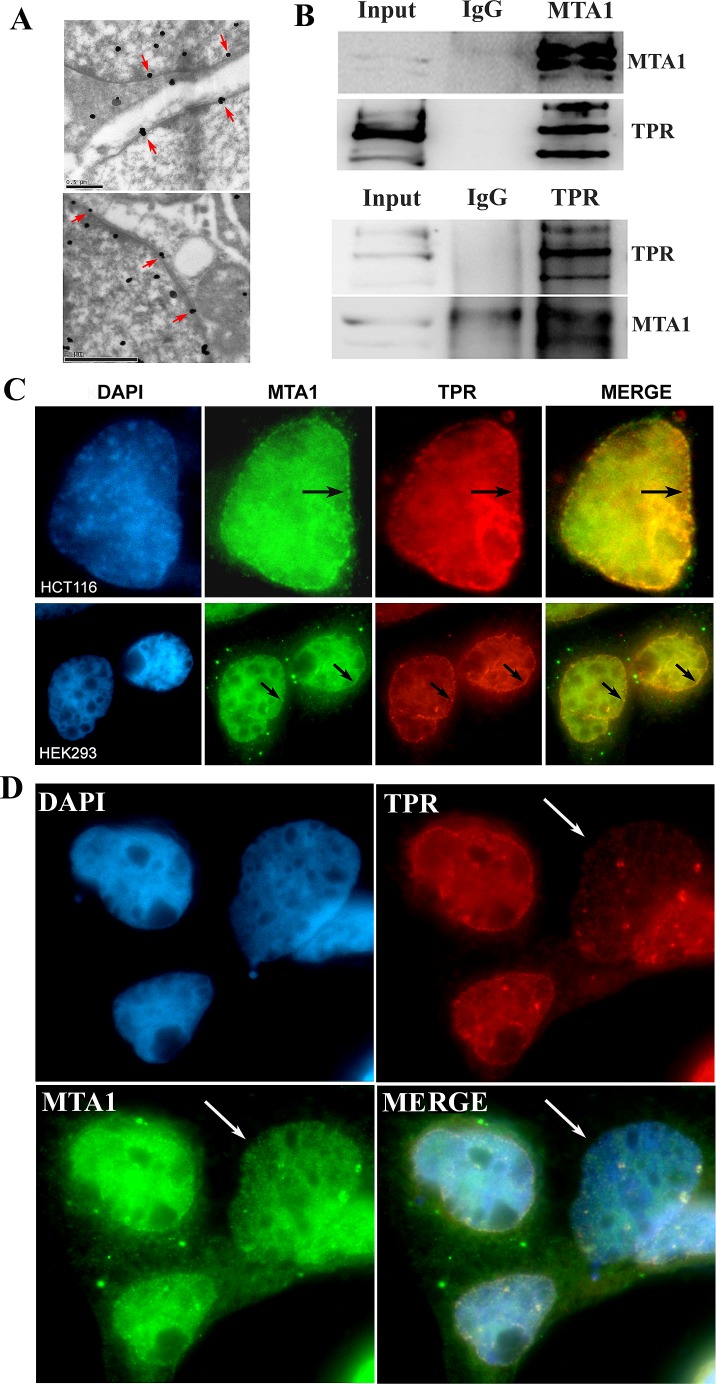
MTA1 interacts and colocalizes with TPR at the nuclear envelope A. The localization of MTA1 on the nuclear envelope (indicated with red arrows) using immuno-electron microscopy; B. Co-IP analysis of the interaction between MTA1 and TPR; C. Immuno-colocalization of MTA1 and TPR at the nuclear envelope of HCT116 and HEK293 cells is indicated with black arrows; D. The knock down of TPR in HEK293 cells impairs the nuclear envelope localization of MTA1. The white arrow indicates a typical TPR knocked down cell.

### MTA1 interacts and colocalizes with nucleoporin TPR on nuclear envelope

The co-IP analysis revealed that MTA1 interacts with nucleoporin TPR (Fig. 5B). Moreover, as shown in Figure 5C, in situ immuno-colocalization analysis also confirmed the colocalization of MTA1 and TPR at the nuclear envelope. TPR is a conserved nucleoporin protein localized within the nuclear basket of the nuclear pore complex[21-24]. Thus, these results suggest the localization of MTA1 at the nuclear pore of the nuclear envelope.

### Localization of MTA1 at the nuclear envelope is dependent on TPR

To determine whether the localization of MTA1 at the nuclear envelope is dependent on TPR, we knocked down TPR expression in HEK293 cells using specific siRNA, and the results showed that in TPR-depleted cells, the localization of MTA1 at the nuclear envelope was also impaired (Fig. 5D). This result suggests that MTA1 is localized at the nuclear envelope in a TPR-dependent manner.

### MTA1 is obviously localized on microtubules in the cytoplasm

**Figure 6:**
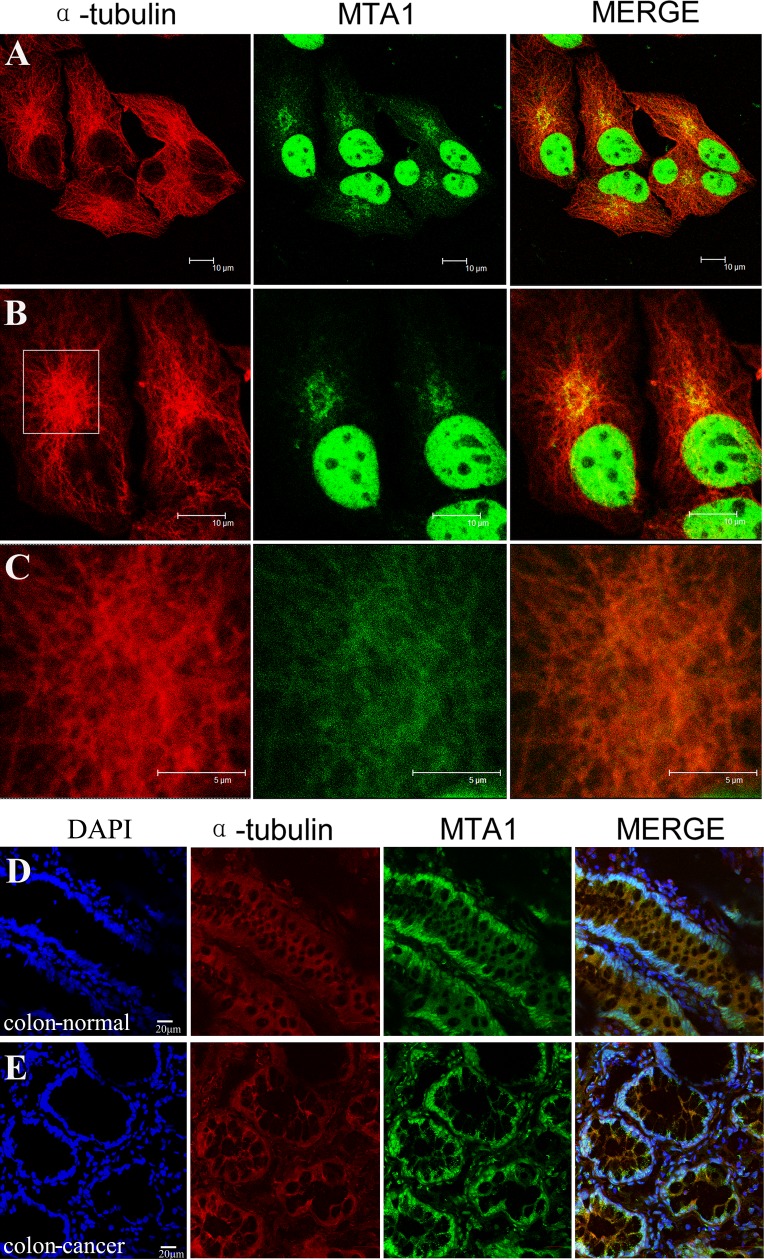
Localization of MTA1 on microtubules in the cytoplasm NCI-H446 cells and colon tissues were immuno-stained with specific antibodies against MTA1 and α-tubulin, followed by detection using a laser scanning confocal microscope. The localization of MTA1 on microtubules in NCI-H446 cells is shown in A, B, and C. An enlargement of the rectangular region in B is shown in C. D and E show the localization of MTA1 on microtubules in the cytoplasm of normal and cancerous colon tissues respectively.

All potential copartners of MTA1 in HCT116 cell lysate were pulled down by an IP technology using two specific antibodies against MTA1 and subsequently identified through mass spectrometry (unpublished data). Many nuclear proteins, including all known NuRD components and cytoplasmic tubulin-associated proteins, and multiple nuclear envelope proteins, including the nucleoporin TPR, were detected. To determine the cytoplasmic localization of MTA1, we co-immunostained NCI-H446 cells (Fig. 6A, 6B and 6C) and colon tissues (Fig. 6D and 6E) with specific antibodies against MTA1 and α-tubulin. The results showed the clear localization of MTA1 on microtubules in the cytoplasm.

### Both nuclear and cytoplasmic MTA1 proteins are involved in cancer progression, however, only nuclear MTA1 inhibits cancer differentiation

Although cytoplasmic localization has been indicated in previous studies, until recently, only the nuclear functions of MTA1 have been examined. Herein, we determined whether cytoplasmic MTA1 is also involved in cancer promotion and attempted to characterize the difference between nuclear and cytoplasmic MTA1 functions.

**Figure 7:**
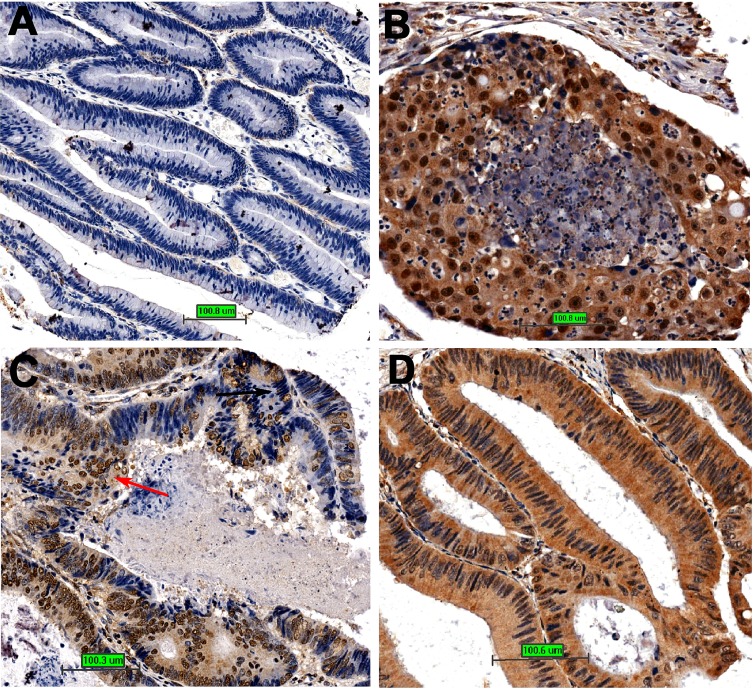
The relationship between MTA1 and cancer differentiation A. Cancer tissue is well differentiated with little MTA1 expression; B, The cancer tissue is poorly differentiated with high nuclear MTA1 expression; C, Different regions with different nuclear MTA1 expression in colon cancer tissue show different differentiation states; D, The cancer tissue is well differentiated with high cytoplasmic but low nuclear MTA1 expression.

Thus, we immunostained a high-density colon cancer microarray, eliminated the invalid tissues, and subsequently scored the intensity of nuclear, cytoplasmic, and overall MTA1 analyses in each tissue. The intensity was graded according to the following rules: 0, no staining; 1, low staining; 2, moderate staining; and 3, strong staining. The correlation between nuclear/cytoplasmic/overall MTA1 intensity and clinicopathological parameters (age, differentiation, clinical stage, T stage, metastasis) was analyzed using SPSS software. As shown in Table 1, in different age groups, no significant difference was detected no matter by nuclear, cytoplasmic or overall MTA1 analysis (P = 0.537, P = 0.302, and P = 0.178, respectively); However, in different groups of clinical stage, T stage and metastasis, the differences are all highly significant by nuclear, cytoplasmic and overall MTA1 analyses (P <0.01 for every test), indicating that both nuclear and cytoplasmic MTA1 were involved in tumor promotion; The role of MTA1 in cancer differentiation is poorly understood. Interestingly, at different stages of cancer differentiation, regardless of cytoplasmic or overall MTA1 analyses, no significant differences were observed (P = 0.527 and P = 0.506, respectively); however, a highly significant difference was detected through nuclear MTA1 analysis (P = 0.000). The differentiation level decreased with increasing nuclear MTA1 expression according to the mean rank, indicating that nuclear MTA1 may inhibit cancer cell differentiation. The different roles for nuclear and cytoplasmic MTA1 in cancer differentiation are well described in Figure 7. In colon cancer with low MTA1 expression, the cancer tissue is well differentiated (Fig. 7A), whereas in colon cancer with high nuclear MTA1 expression, the cancer tissue is poorly differentiated (Fig. 7B). Moreover, as shown in Figure 7C, this tendency was also observed in cancer tissues with varying differentiation levels as follows: regions with high nuclear MTA1 are poorly differentiated (indicated with the red arrow), and regions with low nuclear MTA1 expression are well differentiated (indicated with the black arrow). However, in colon cancer tissues with high cytoplasmic but low nuclear MTA1 expression (the overall MTA1 level is also high), the cancer tissue is still well differentiated (Fig. 7D), indicating an ineffective role for cytoplasmic MTA1 in the regulation of cancer differentiation.

**Table 1:** Analysis of the correlations between nuclear, cytoplasmic, and overall MTA1 with the clinicopathological parameters of colon cancer

	Total	Nuclear Intensity				Mean rank	P value	Cytoplasmic Intensity				Mean rank	P value	Overall Intensity				Mean rank	P value
		0	1	2	3			0	1	2	3			0	1	2	3		
Age, years																			
≤60	153	10	58	71	14	136.83	0.537	8	73	50	22	138.37	0.302	9	63	60	21	139.62	0.178
>60	115	10	43	55	7	131.40		5	60	44	6	129.36		8	53	46	8	127.69	
Differen tiation																			
Well	28	8	16	4	0	69.93	0.000	3	15	5	5	123.61	0.527	3	12	7	6	134.38	0.506
Moderate	190	11	68	99	12	138.45		7	93	70	20	137.49		12	76	84	18	137.28	
Poor	50	1	17	23	9	155.66		3	25	19	3	129.22		2	28	15	5	123.99	
Clinical stage																			
I	22	9	13	0	0	46.25	0.000	5	15	2	0	73.73	0.000	10	10	2	0	55.36	0.000
II	218	11	77	111	19	142.04		8	101	81	28	141.91		7	85	97	29	146.50	
III	21	0	6	13	2	159.07		0	13	8	0	123.24		0	16	5	0	101.93	
IV	7	0	5	2	0	103.43		0	4	3	0	128.64		0	5	2	0	107.21	
Tumor status																			
T1	4	1	3	0	0	55.88	0.000	1	3	0	0	61.75	0.003	2	2	0	0	42.25	0.000
T2	20	8	10	2	0	58.15		4	12	4	0	88.10		8	8	4	0	71.10	
T3	156	8	60	74	14	138.52		8	71	54	23	141.27		6	60	68	22	146.50	
T4	88	3	28	50	7	148.30		0	47	36	5	136.35		1	46	34	7	131.83	
Metastasis																			
N0M0	191	18	80	82	11	124.80	0.000	12	102	68	9	124.04	0.000	16	83	81	11	127.28	0.009
N1-N2,NOM1	77	2	21	44	10	158.57		1	31	26	19	160.44		1	33	25	18	152.40	
Total	268	20	101	126	21			13	133	94	28			17	116	106	29		
Mann-Whitney U test for age and metastasis analysis																			
Kruskal-Wallis H (K) test for differentiation, clinical stage and tumor status analysis																			

### Overexpression of MTA1 in HCT116 cells inhibited differentiation and promoted proliferation, whereas MTA1 knockdown resulted in cell differentiation and death

**Figure 8:**
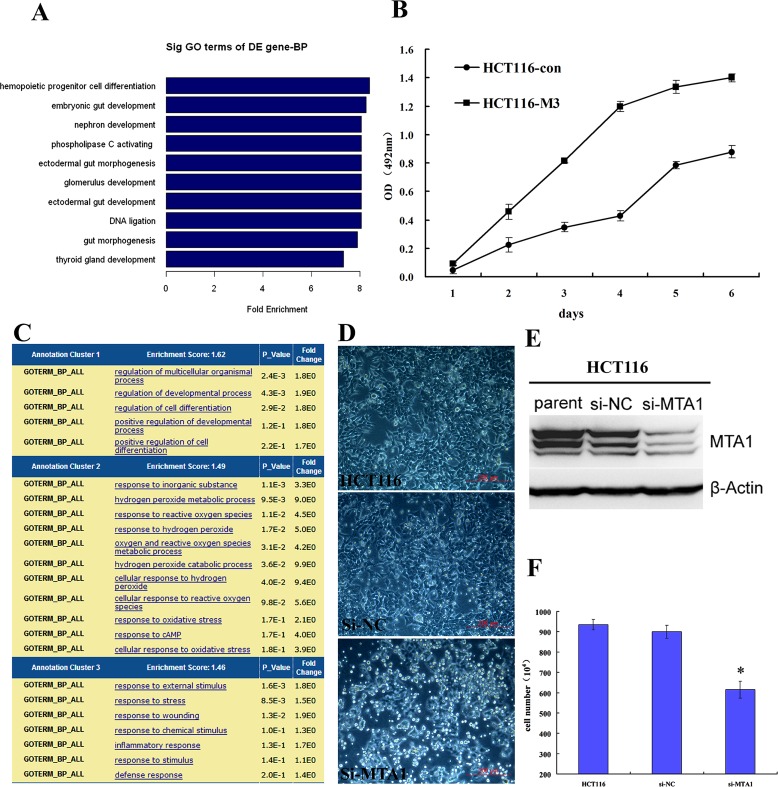
Role of MTA1 in cancer differentiation A. The genes down-regulated in response to MTA1 overexpression are highly enriched on gut development and differentiation; B. MTA1 promotes the proliferation of cancer cells; C. The genes up-regulated in response to MTA1 knockdown show the highest enrichment score on the functional annotation cluster for development and differentiation; D. Transient knock down of MTA1 using siRNA causes the floating and death of HCT116 colon cancer cells; E. Western blot analysis showing the effect of MTA1 siRNA (si-MTA1) on HCT116 cells; F. At 72 h after transfection, the number of survival si-MTA1 transfected cells was 2/3 that of control cells (P<0.05).

To further determine the role for MTA1 in cancer differentiation, we utilized expression profile chip analysis to compare the genome features between HCT116 control and MTA1-overexpressing HCT116-M3 cells. The GO analysis revealed that the genes down-regulated in response to MTA1 overexpression are highly enriched on gut development and differentiation (Fig. 8A), which was not observed in the analysis of the up-regulated genes, indicating that MTA1 overexpression induced a less differentiated genome state. Moreover, MTT analysis showed that cell proliferation was significantly promoted after MTA1 overexpression (Fig. 8B).

Furthermore, when MTA1 was transiently knocked down in HCT116 cells using specific siRNA (Fig. 8E), the GO analysis of the up-regulated genes detected through expression profile sequencing showed the highest enrichment score on the functional annotation cluster for development and differentiation (Fig. 8C). This result was not observed in the down-regulated gene analysis, indicating that MTA1 deletion induced a more differentiated genome status. In the siMTA1-transfected group, floating and cell death were obviously observed in a large proportion of cells (Fig. 8D). At 72 h after transfection, the number of surviving siMTA1-transfected cells was only 2/3 that of the control cells (Fig. 8F).

## DISCUSSION

MTA1 is a metastasis-promoting gene that encodes a protein comprising typical DNA-binding motifs and nuclear localization signals. Initial studies have reported that MTA1 was exclusively localized in the nucleus[16]. However, recent studies using immunohistochemistry indicate that MTA1 may also be distributed in the cytoplasm. To further explore the function and mechanism of MTA1, we examined the subcellular distribution of this protein using multiple molecular approaches. We examined both endogenous and exogenous MTA1 and provided the first evidence that MTA1 is localized in the nucleus, cytoplasm, and nuclear envelope.

We observed the general expression of MTA1 in virtually all tissues and cell lines, regardless of normal or cancerous or adult or embryonic, suggesting an important physiological role for MTA1, in addition to its well-known roles in cancer. MTA1 expression is typically lower in normal adult tissues, except the brain, liver, kidney, and cardiac muscle. In embryonic tissues, MTA1 is highly expressed in nerve tissues, such as the brain, eyes, and spinal cord, indicating a potentially important role for MTA1 in nerve development. The majority of MTA1 is localized in the nucleus of adult normal tissues, except for skeletal and cardiac muscle. In contrast, MTA1 was primarily localized to the cytoplasm in embryonic tissues. Considering that nuclear MTA1 inhibits cell differentiation, embryonic differentiation may require MTA1 to be cytoplasm-positioned

The localization of MTA1 at the nuclear envelope was directly visualized using both fluorescence and electron microscopy, and this localization was further confirmed through interaction and colocalization with the nucleoporin protein TPR. Knockdown of TPR disrupts the localization of MTA1 at the nuclear envelope. TPR is a conserved nucleoporin protein located in the nuclear basket of the nuclear pore complex, and TPR mediates the nuclear export of macromolecules[21-24]. These results suggest that MTA1 localizes to nuclear pores on the nuclear envelope in a TPR-dependent manner.

The identification of MTA1 copartners revealed the localization of MTA1 on microtubules in the cytoplasm. Microtubules are important components of the cytoskeleton and function in a number of movement-related cellular processes, such as cell mobility, which is particularly high in metastatic cancer cells. The colon cancer tissue array analysis revealed that cytoplasmic MTA1 is also significantly associated with tumor metastasis (p<0.001). Whether MTA1 regulates the metastasis of cancer cells through microtubules should be further investigated.

In a recent report[25], Aramaki et al. demonstrated that MTA1 directly interacts with the cytoplasmic protein endophilin 3; however, these results do not provide conclusive evidence for the involvement of MTA1 in the regulation of endophilin 3-mediated endocytosis because in this previous study, MTA1 and endophilin 3 were localized to different compartments of the cell (i.e., the nucleus and cytoplasm). In the present study, we showed the cytoplasmic localization of MTA1, which is consistent with the reported interaction with endophilin 3, and provide evidence for the potential function of cytoplasmic MTA1 in endocytosis regulation. Indeed, microtubules play essential roles in endocytosis regulation[26].

Nuclear MTA1 binds to DNA and regulates gene transcription through chromatin remodeling[27-29]. In the present study, we showed that cytoplasmic MTA1 is localized on microtubules with unexplored functions. Although both nuclear and cytoplasmic MTA1 are involved in promoting cancer malignancies, such as tumor stage and metastasis, the different subcellular locations and interacting partners may confer different functions. For example, nuclear but not cytoplasmic MTA1 is significantly associated with tumor differentiation (P = 0.000 and P = 0.527, respectively), a novel role demonstrated in the present study. Thus, the results of this study suggest a new subcellular localization of MTA1, indicating a potential novel function for this protein in the cytoplasm, particularly in association with microtubules.

## MATERIALS AND METHODS

### Cell culture

Both cancer cell lines (colon cancer cell line HCT116, small cell lung cancer cell line NCI-H446, endometrial cancer cell line Ishikawa, brain cancer cell line SF-767, hepatocellular carcinoma cell line Hep3B, colon cancer cell line HCT8, and cervical cancer cell line Caski) and normal cell lines (human embryonic kidney cell line HEK293 and normal skin cell line HaCaT) were used in this study. Stably transfected MTA1-flag overexpressing cell lines HCT116-M1 and HCT116-M3 were constructed through lentiviral infection of HCT116 cells. All of the cell lines were grown in DMEM supplemented with 10% fetal bovine serum at 37°C in a 5% CO2 standard incubator.

### Antibodies

The following antibodies were used: mouse monoclonal antibody anti-MTA1 (Abcam); rabbit polyclonal antibody anti-MTA1 (Abcam); mouse monoclonal antibody anti-HDAC2 (Abcam); rabbit polyclonal antibody anti- HDAC2 (Bioworld Technology); rabbit polyclonal antibody anti-GAPDH (Bioworld Technology); rabbit polyclonal antibody anti-histone H3 (Bioworld Technology); and rabbit polyclonal antibody anti-TPR (Santa Cruz).

### Tissue microarrays, mouse embryos and immunohistochemistry

The mouse normal tissue chip (MO1601), human normal tissue chip (FDA999), and high-density colon cancer tissue microarray (CO6161) were obtained from the Alenabio Company. Day 14 mouse embryos were fixed with 4% paraformaldehyde and embedded in paraffin blocks. Immunohistochemistry was performed using indirect horseradish peroxidase staining to detect the expression and distribution of MTA1 in normal and cancer tissues using a mouse monoclonal antibody against MTA1 (1:1000).

### Immunofluorescence

For IF experiments, the cells were grown on sterilized cover slips and subsequently fixed in 4% paraformaldehyde at room temperature for 15 min, washed twice in PBS, permeabilized with 0.25% Triton X-100 at room temperature for 10 min, and washed twice in PBS before incubation in PBS containing 0.5% bovine serum albumin for 30 min. The cover slips were subsequently incubated overnight in primary antibody, followed by a 1 h incubation in secondary antibody diluted in blocking buffer. The cover slips were mounted with mounting medium containing DAPI. The images were acquired using fluorescence (Olympus) or confocal laser scanning (Leica) microscopy.

### Protein extraction and Western blot

Whole cell lysate was prepared with RIPA buffer (Beyotime Biotech, China); Cytoplasmic and nuclear extracts were prepared using Nuclear and Cytoplasmic Protein Extraction kit (Beyotime Biotech, China) according to the manufacturer's instructions. Protein concentrations were quantified using a BCA assay (Sigma-Aldrich), and 80-100 μg protein was used per lane; Western blot analysis was carried out according to standard procedures.

### Immunoprecipitation

For the immunoprecipitation (IP) analysis, 1 mg of cell lysate was incubated with 1-2 μg of primary antibody overnight at 4°C on a rocker platform, followed by incubation with 25 μl of protein A/G PLUS-agarose (Santa Cruz) for 2 h at 4°C. The immunoprecipitates were washed 3 times with NP-40 buffer (50 mM Tris-HCl, pH 8.0, 0.5% NP-40, 10% Glycerol, 150 mM NaCl, 2 mM MgCl_2_, and 1 mM EDTA, with protease inhibitors) and collected after centrifugation at 6000 rpm for 5 min. Subsequently, the beads were boiled in SDS loading buffer for 10 min for Western blot analysis.

### In Situ PLA detection

In situ PLA experiments were performed to visualize the interaction of MTA1 and HDAC2 in cells using the Duolink kit (Olink Biosciences AB) as previously described[30]. The cover slips were incubated with primary antibodies (mouse anti-MTA1 and Rabbit anti-HDAC2) at room temperature for 1 h, washed 3 times for 5 min in PBS containing 0.1% Tween 20. Secondary proximity probes (Mouse- PLUS and Rabbit- MINUS) were incubated for 2 h at 37°C. The cells were washed 3 times for 5 min in PBS containing 0.1% Tween 20 at 37°C. All subsequent steps were performed according to the Duolink kit protocol (Olink Biosciences AB). The images were acquired using fluorescence microscopy (Olympus).

### Immunogold electron microscopy

The specimens for the immuno-electron microscopy of cultured HCT116 cells were prepared in a manner similar to that previously described[31]. After fixation with 4% formaldehyde and 0.05% glutaraldehyde in PBS for 5 min, the cells were permeabilized with 0.25% Triton X-100, blocked with 5% goat serum, and subsequently processed for gold labeling. The specimens were analyzed at 60 or 80 kV using transmission electron microscopy.

### Gene expression profile analysis through chip or sequencing and gene ontology analyses

A gene expression profile chip analysis (NimbleGen, 12x135K microarray) was conducted to compare the gene expression profiles between HCT116-control and MTA1-overexpressing HCT116-M3 cells. Gene expression profile sequencing was performed to compare the gene expression profiles between control HCT116-si-NC cells and MTA1 knockdown HCT116-si-MTA1 cells. A gene ontology analysis was performed using the Database for Annotation, Visualization, and Integrated Discovery 6.7 (DAVID 6.7, http://david.abcc.ncifcrf.gov/home.jsp)[32]with GOTERM annotation (GOTERM_BP_ALL).

### Statistical Analysis

All statistical analyses were performed using SPSS 13.0 statistical software. The Mann-Whitney U test was used for age and metastasis analyses, and the Kruskal-Wallis H(K) test was used for differentiation, clinical stage, and tumor status analyses. Differences were considered significant at P-value < 0.05.

## References

[1] Toh Y, Pencil SD, Nicolson GL (1994). A novel candidate metastasis-associated gene, mta1, differentially expressed in highly metastatic mammary adenocarcinoma cell lines. cDNA cloning, expression, and protein analyses. J Biol Chem.

[2] Toh Y, Pencil SD, Nicolson GL (1995). Analysis of the complete sequence of the novel metastasis-associated candidate gene, mta1, differentially expressed in mammary adenocarcinoma and breast cancer cell lines. Gene.

[3] Pencil SD, Toh Y, Nicolson GL (1993). Candidate metastasis-associated genes of the rat 13762NF mammary adenocarcinoma. Breast Cancer Res Treat.

[4] Toh Y, Nicolson GL (2009). The role of the MTA family and their encoded proteins in human cancers: molecular functions and clinical implications. Clin Exp Metastasis.

[5] Xue Y, Wong J, Moreno GT, Young MK, Cote J, Wang W (1998). NURD, a novel complex with both ATP-dependent chromatin-remodeling and histone deacetylase activities. Mol Cell.

[6] Zhang Y, LeRoy G, Seelig HP, Lane WS, Reinberg D (1998). The dermatomyositis-specific autoantigen Mi2 is a component of a complex containing histone deacetylase and nucleosome remodeling activities. Cell.

[7] Mazumdar A, Wang RA, Mishra SK, Adam L, Bagheri-Yarmand R, Mandal M, Vadlamudi RK, Kumar R (2001). Transcriptional repression of oestrogen receptor by metastasis-associated protein 1 corepressor. Nat Cell Biol.

[8] Balasenthil S, Gururaj AE, Talukder AH, Bagheri-Yarmand R, Arrington T, Haas BJ, Braisted JC, Kim I, Lee NH, Kumar R (2007). Identification of Pax5 as a target of MTA1 in B-cell lymphomas. Cancer Res.

[9] Gururaj AE, Singh RR, Rayala SK, Holm C, den Hollander P, Zhang H, Balasenthil S, Talukder AH, Landberg G, Kumar R (2006). MTA1, a transcriptional activator of breast cancer amplified sequence 3. Proc Natl Acad Sci U S A.

[10] Pakala SB, Singh K, Reddy SD, Ohshiro K, Li DQ, Mishra L, Kumar R (2011). TGF-beta1 signaling targets metastasis-associated protein 1, a new effector in epithelial cells. Oncogene.

[11] Pakala SB, Rayala SK, Wang RA, Ohshiro K, Mudvari P, Reddy SD, Zheng Y, Pires R, Casimiro S, Pillai MR, Costa L, Kumar R (2013). MTA1 promotes STAT3 transcription and pulmonary metastasis in breast cancer. Cancer Res.

[12] Li DQ, Pakala SB, Nair SS, Eswaran J, Kumar R (2012). Metastasis-associated protein 1/nucleosome remodeling and histone deacetylase complex in cancer. Cancer Res.

[13] Kumar R, Wang RA, Bagheri-Yarmand R (2003). Emerging roles of MTA family members in human cancers. Semin Oncol.

[14] Manavathi B, Kumar R (2007). Metastasis tumor antigens, an emerging family of multifaceted master coregulators. J Biol Chem.

[15] Yao YL, Yang WM (2003). The metastasis-associated proteins 1 and 2 form distinct protein complexes with histone deacetylase activity. J Biol Chem.

[16] Nawa A, Nishimori K, Lin P, Maki Y, Moue K, Sawada H, Toh Y, Fumitaka K, Nicolson GL (2000). Tumor metastasis-associated human MTA1 gene: its deduced protein sequence, localization, and association with breast cancer cell proliferation using antisense phosphorothioate oligonucleotides. J Cell Biochem.

[17] Moon WS, Chang K, Tarnawski AS (2004). Overexpression of metastatic tumor antigen 1 in hepatocellular carcinoma: Relationship to vascular invasion and estrogen receptor-alpha. Hum Pathol.

[18] Balasenthil S, Broaddus RR, Kumar R (2006). Expression of metastasis-associated protein 1 (MTA1) in benign endometrium and endometrial adenocarcinomas. Hum Pathol.

[19] Li W, Ma L, Zhao J, Liu X, Li Z, Zhang Y (2009). Expression profile of MTA1 in adult mouse tissues. Tissue Cell.

[20] Simpson A, Uitto J, Rodeck U, Mahoney MG (2001). Differential expression and subcellular distribution of the mouse metastasis-associated proteins Mta1 and Mta3. Gene.

[21] Cordes VC, Reidenbach S, Rackwitz HR, Franke WW (1997). Identification of protein p270/Tpr as a constitutive component of the nuclear pore complex-attached intranuclear filaments. J Cell Biol.

[22] Shah S, Tugendreich S, Forbes D (1998). Major binding sites for the nuclear import receptor are the internal nucleoporin Nup153 and the adjacent nuclear filament protein Tpr. J Cell Biol.

[23] Frosst P, Guan T, Subauste C, Hahn K, Gerace L (2002). Tpr is localized within the nuclear basket of the pore complex and has a role in nuclear protein export. J Cell Biol.

[24] Krull S, Thyberg J, Bjorkroth B, Rackwitz HR, Cordes VC (2004). Nucleoporins as components of the nuclear pore complex core structure and Tpr as the architectural element of the nuclear basket. Mol Biol Cell.

[25] Aramaki Y, Ogawa K, Toh Y, Ito T, Akimitsu N, Hamamoto H, Sekimizu K, Matsusue K, Kono A, Iguchi H, Takiguchi S (2005). Direct interaction between metastasis-associated protein 1 and endophilin 3. FEBS Lett.

[26] Soldati T, Schliwa M (2006). Powering membrane traffic in endocytosis and recycling. Nat Rev Mol Cell Biol.

[27] Xue Y, Wong J, Moreno GT, Young MK, Cote J, Wang W (1998). NURD, a novel complex with both ATP-dependent chromatin-remodeling and histone deacetylase activities. Mol Cell.

[28] Molli PR, Singh RR, Lee SW, Kumar R (2008). MTA1-mediated transcriptional repression of BRCA1 tumor suppressor gene. Oncogene.

[29] Salot S, Gude R (2013). MTA1-mediated transcriptional repression of SMAD7 in breast cancer cell lines. Eur J Cancer.

[30] Jarvius M, Paulsson J, Weibrecht I, Leuchowius KJ, Andersson AC, Wahlby C, Gullberg M, Botling J, Sjoblom T, Markova B, Ostman A, Landegren U, Soderberg O (2007). In situ detection of phosphorylated platelet-derived growth factor receptor beta using a generalized proximity ligation method. Mol Cell Proteomics.

[31] Cordes VC, Reidenbach S, Rackwitz HR, Franke WW (1997). Identification of protein p270/Tpr as a constitutive component of the nuclear pore complex-attached intranuclear filaments. J Cell Biol.

[32] Huang dW, Sherman BT, Lempicki RA (2009). Systematic and integrative analysis of large gene lists using DAVID bioinformatics resources. Nat Protoc.

